# Transcriptome analysis of long non-coding RNA and mRNA Profiles in VSV-infected BHK-21 Cells

**DOI:** 10.1186/s12864-024-09991-9

**Published:** 2024-01-15

**Authors:** Wuweiyi Han, Xiaojuan Fei, Fan Yang, Xintong Sun, Jianshe Yang, Jinxin Qiu, Luhua Zhang, Wenhui Zhang, Guohua Chen, Wei Han, Xiaobo He, Yongsheng Liu, Weike Li

**Affiliations:** 1 Hebei Key Laboratory of Preventive Veterinary Medicine, College of Animal Science and Technology, Hebei Normal University of Science &Technology, Qinhuangdao, 066004 China; 2 State Key Laboratory for Animal Disease Control and Prevention, College of Veterinary Medicine, Lanzhou University, Lanzhou Veterinary Research Institute, Chinese Academy of Agricultural Sciences, Lanzhou, 730000 China; 3 Center of Gansu Provincial Vaccine Engineering Research, Lanzhou Institute of Biological Products, Lanzhou, 730046 Gansu Province China; 4 Shandong Zhuohua Biotechnology Co., Ltd, Liaocheng, 252126 China

**Keywords:** Vesicular stomatitis virus, RNA-Seq, LncRNA, mRNA, Differentially expressed genes

## Abstract

**Background:**

Vesicular stomatitis virus (VSV) is a typical non-segmented negative-sense RNA virus of the genus *Vesiculovirus* in the family *Rhabdoviridae*. VSV can infect a wide range of animals, including humans, with oral blister epithelial lesions. VSV is an excellent model virus with a wide range of applications as a molecular tool, a vaccine vector, and an oncolytic vector. To further understand the interaction between VSV and host cells and to provide a theoretical basis for the application prospects of VSV, we analyzed the expression of host differentially expressed genes (DEGs) during VSV infection using RNA-Seq.

**Results:**

Our analyses found a total of 1015 differentially expressed mRNAs and 161 differentially expressed LncRNAs in BHK-21 cells infected with VSV for 24 h compared with controls. Gene Ontology and Kyoto Encyclopedia of Genes and Genomes enrichment showed that the differentially expressed lncRNAs and their target genes were mainly concentrated in pathways related to apoptosis, cancer, disease, and immune system activation, including the TNF, P53, MAPK, and NF-kappaB signaling pathways. The differentially expressed lncRNA can modulate immune processes by regulating genes involved in these signaling transmissions. Ten randomly selected DEGs, namely, *Il12rb2*, *F2*, *Masp2*, *Mcl1, FGF18*, *Ripk1*, *Fas*, *BMF*, *POLK*, and *JAG1*, were validated using RT-qPCR. As predicted through RNA-Seq analysis, these DEGs underwent either up- or downregulation, suggesting that they may play key regulatory roles in the pathways mentioned previously.

**Conclusions:**

Our study showed that VSV infection alters the host metabolic network and activates immune-related pathways, such as MAPK and TNF. The above findings provide unique insights for further study of the mechanism of VSV–host interactions and, more importantly, provide a theoretical basis for VSV as an excellent vaccine carrier.

**Supplementary Information:**

The online version contains supplementary material available at 10.1186/s12864-024-09991-9.

## Background

Vesicular stomatitis virus (VSV), a typical non-segmented, negative-sense RNA virus, belongs to the *Vesiculovirus* genus of the family *Rhabdoviridae* [1]. Based on antibody neutralization tests and complement binding tests, VSV is divided into the New Jersey and Indiana serotypes. The genome of VSV encodes five viral proteins: nucleocapsid (N), phosphoprotein (P), matrix (M), glycoprotein (G), and large protein (L) [2]. The virus has a broad host range such as horses, cattle, swine, goats, rodents, and humans [3]. Infected horses, cattle, and pigs can develop oral vesicular epithelial lesions [3]. Vesicular stomatitis was first described after an outbreak in the USA in 1916. VSV is now considered endemic in parts of equatorial America and in the southwestern states of the USA, both of which witness outbreaks every 10 years [4]. Currently, several aspects of VSV transmission are not well understood.

VSV’s genome is of appropriate size, and it can insert 4–5 kb of exogenous genes [5]. It can express unrelated glycoproteins on the viral surface. In addition, VSV can infect a wide range of cell lines, where it rapidly replicates to produce large numbers of infectious viral particles. Therefore, VSV is considered a model virus that can serve as a good molecular tool and vaccine vector. The realization of these applications often requires the use of reverse genetic systems, which allow viruses to be modified at the genetic level. In 1995, Whela et al. succeeded in rescuing infectious VSV particles from a full-length cDNA clone of the viral genome [6]. The successful establishment of the VSV reverse genetic system has opened up the possibility of manipulating the VSV genome and therefore provided a basis for the development of VSV as a widely used research tool, vaccine platform, and oncolytic vector.

Long-stranded non-coding RNA (lncRNA) is a class of non-coding RNAs larger than 200 nucleotides in length, which are potentially involved in the development of human diseases, such as cancer [7]. In viral infections, lncRNAs play an important role in the genetic stabilization of viruses and also affect the generation of innate immune responses in the host cells after viral infection [8]. For example, lncRNA-Acod1 (an lncRNA identified by its most recent encoding gene Acod1, aconitate decarboxylase 1) can be induced by a variety of viruses but not be induced by type I interferon. It promotes viral replication in mouse and human cells [9]. Host lncRNAs also play an important role in the process of virus replication. It has been suggested that the pseudogene-derived lncRNA PCNAP1 and its ancestor PCNA may regulate hepatitis B virus replication and promote hepatocarcinogenesis [10]. In addition, host lncRNAs work together with other non-coding RNAs to influence viral replication. The various types of RNAs involved in the complex network of transcriptional regulation in organisms include competitive endogenous RNAs (ceRNAs), such as mRNA, lncRNA, and circRNA, all of which can bind competitively with miRNAs and together influence the replication process of viruses. Most of the circRNAs are derived from pre-mRNAs, which are clipped to form circRNAs. CircRNAs can competitively bind miRNAs with LncRNAs and mRNAs, resulting in regulation of lncRNAs or mRNAs. lncRNA and mRNA can sometimes have similar sequences; therefore, lncRNA can be used as ceRNA to trap miRNA through similar sequences and release mRNA to perform normal biological functions. In a study by Li et al., qPCR of 144 clinical sputum specimens showed that lncRNA NRAV expression was significantly lower in respiratory syncytial virus (RSV)-positive patients than in uninfected patients and that NRAV overexpression promoted RSV production in vitro, suggesting that reduced NRAV in RSV infection is part of the host's antiviral response. Further studies revealed that NRAV competes with the vesicle protein Rab5c for the microRNA miR509-3p in the cytoplasm to promote RSV vesicle translocation and accelerate RSV proliferation [11].

Currently, there are very few studies related to the lncRNAs with VSV. In our experiment, VSV was used to infect BHK-21 cells, and the cells were collected 24 h later for transcriptome sequencing. The changes in the expression profiles of lncRNA and mRNA in VSV-infected host cells and their association were analyzed to screen potential candidate lncRNAs and target genes in VSV infection. Currently, there are no VSV-related lncRNA studies; therefore, the results of this experiment may provide new research ideas and drug targets for the prevention and treatment of VSV.

## Results

### Growth curves of VSV

**Fig. 1:**
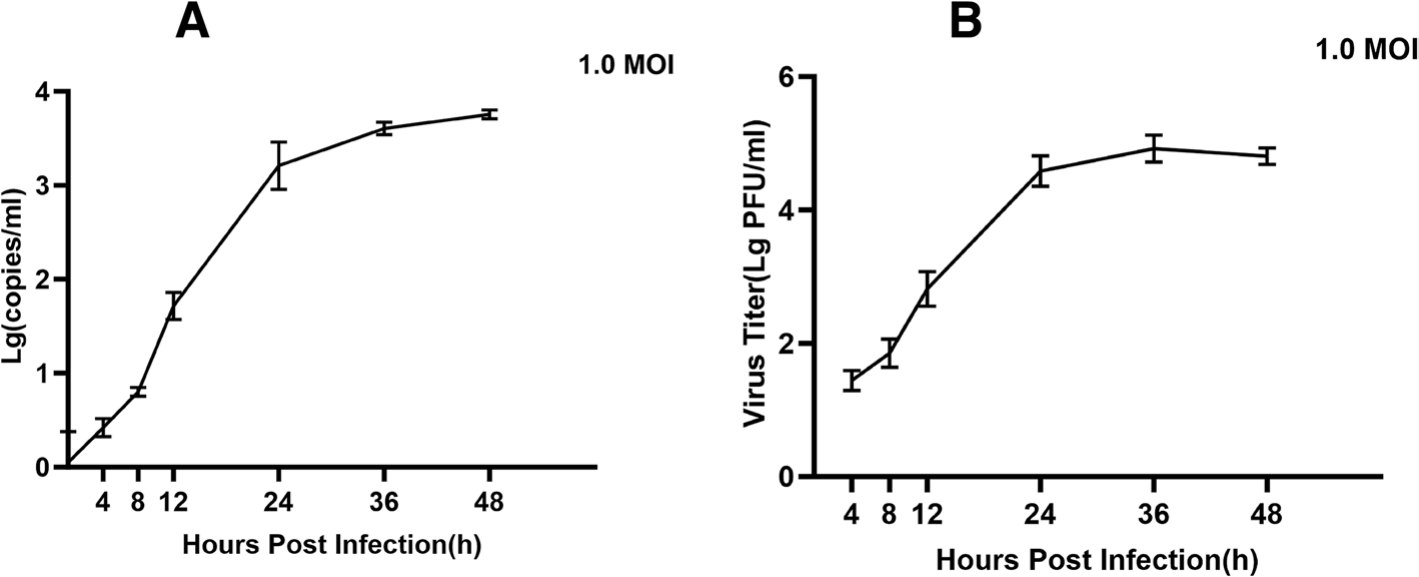
Growth curves of VSV in BHK-21 cells. The viral titer at different times was detected using RT-qPCR, and it similarly stabilized after 24 h (**A**). The plaque assay was used to detect the viral titer at different times, which stabilized after 24 h (**B**). The error bar indicates standard deviation

To determine the intracellular replication cycle, the one-step growth curve method was used to define the dynamics of VSV infection on BHK-21 cells. Because all stages of the VSV life cycle were observed within 48 h, we chose to infect the cells at the value of 1.0 MOI and collect the supernatants at 4, 8, 12, 24, 36, and 48 h. To ensure more reliable results, we used two methods to plot the one-step growth curves. First, the growth curve of VSV was determined using RT-qPCR to detect the changes in the VSV genome copy number in the cell supernatant at 4, 8, 12, 24, 36, and 48 h. The growth curves showed that the amount of virus in the cell supernatant remained essentially stable for 8 h after infection, after which the viral load in the cell supernatant increased rapidly. The viral copy number peaked at 24 h and then remained relatively stable until 48 h (Fig. 1a). The second method was to determine the titer of the virus in the supernatant as described previously using plaque assays and to plot the growth curve. The plaque assays showed similar results; that is, the viral titer remained essentially stable until 8 h, peaked at around 24 h, and subsequently remained stable until 48 h (Fig. 1b). As shown in the results above, we chose 1.0 MOI of BHK-21 cells infected with VSV for 24 h and then collected the cells for subsequent sequencing of the transcriptome.

### Evaluation of the transcriptome sequencing data

The quality of the raw reads obtained by sequencing was assessed, and to obtain high-quality clean reads, the reads were further filtered using fastp (version 0.18.0). We checked the sequencing error rate and obtained 99.32–99.44% clean reads for subsequent analysis. The Q30 percentages of the clean data for all samples were higher than 90.94%, and the GC content of the clean data for all samples ranged between 51.68% and 54.05%. The reads were compared with the rRNA database using the Bowtie2 software (version 2.2.8). The percentage of mapped reads after removal of rRNA was from 99.90% to 99.94%. For further analysis, high-quality pure reads were mapped to the reference *Mesocricetus auratus* genome (Ensembl_release104) using HISAT 2.2.4. Approximately 51.67–73.40% of the clean reads were successfully mapped to the reference *Mesocricetus auratus* genome (Table 1). Since transcriptome sequencing will fragment the mRNA before reverse transcription. Therefore, the raw data from sequencing is required assembly of the reads to explore new genes and new splice variants. The mapped reads of each sample were assembled using StringTie v1.3.1 in a reference-based approach. In total, 20,387 genes were assembled from the sequenced sequences, of which 12,547 were successfully localized to the *Mesocricetus auratus* reference genome, and 7840 new genes were predicted. The transcripts obtained above were used for the subsequent experiments.

**Table 1:** Data quality control and sequence comparison analysis

Sample	Adapter (%)	LowQuality (%)	polyA (%)	N (%)	Filtering of clean reads (%)	BF_Q30 (%)	BF_GC (%)	rRNA Mapped_Reads (%)	rRNA Unmapped_Reads (%)	Total_Mapped (%)
BVV-1	11,642 (0.11%)	47,778 (0.45%)	0 (0.00%)	0 (0.00%)	10,556,526 (99.44%)	1,461,866,815 (91.80%)	837,766,608 (52.61%)	7312 (0.07%)	10,549,214 (99.93%)	5,454,910 (51.67%)
BVV-2	11,406 (0.11%)	54,702 (0.52%)	0 (0.00%)	520 (0.00%)	10,423,434 (99.36%)	1,434,020,347 (91.14%)	831,891,697 (52.87%)	7754 (0.07%)	10,415,680 (99.93%)	5,699,600 (54.68%)
BVV-3	11,732 (0.10%)	53,468 (0.47%)	0 (0.00%)	630 (0.01%)	11,256,992 (99.42%)	1,549,855,192 (91.25%)	877,697,944 (51.68%)	10,968 (0.10%)	11,246,024 (99.90%)	5,868,299 (52.13%)
BVV-4	12,236 (0.11%)	51,996 (0.46%)	0 (0.00%)	606 (0.01%)	11,304,776 (99.43%)	1,562,909,022 (91.64%)	889,486,395 (52.16%)	7118 (0.06%)	11,297,658 (99.94%)	5,949,614 (52.63%)
Mock-1	16,876 (0.09%)	89,846 (0.47%)	0 (0.00%)	640 (0.00%)	18,834,154 (99.43%)	2,621,870,692 (92.28%)	1,535,555,933 (54.05%)	11,472 (0.06%)	18,822,682 (99.94%)	13,438,746 (71.35%)
Mock-2	12,760 (0.08%)	99,900 (0.60%)	0 (0.00%)	610 (0.00%)	16,629,940 (99.32%)	2,302,391,724 (91.67%)	1,356,380,040 (54.01%)	12,570 (0.08%)	16,617,370 (99.92%)	12,082,627 (72.66%)
Mock-3	13,418 (0.08%)	97,384 (0.58%)	0 (0.00%)	544 (0.00%)	16,683,394 (99.34%)	2,315,473,276 (91.91%)	1,346,251,150 (53.44%)	12,700 (0.08%)	16,670,694 (99.92%)	12,245,847 (73.40%)
Mock-4	16,150 (0.09%)	103,964 (0.57%)	0 (0.00%)	628 (0.00%)	18,117,724 (99.34%)	2,487,859,109 (90.94%)	1,458,325,799 (53.31%)	13,330 (0.07%)	18,104,394 (99.93%)	13,172,252 (72.70%)

### Expression levels of the genes and differential expression analysis

**Fig. 2:**
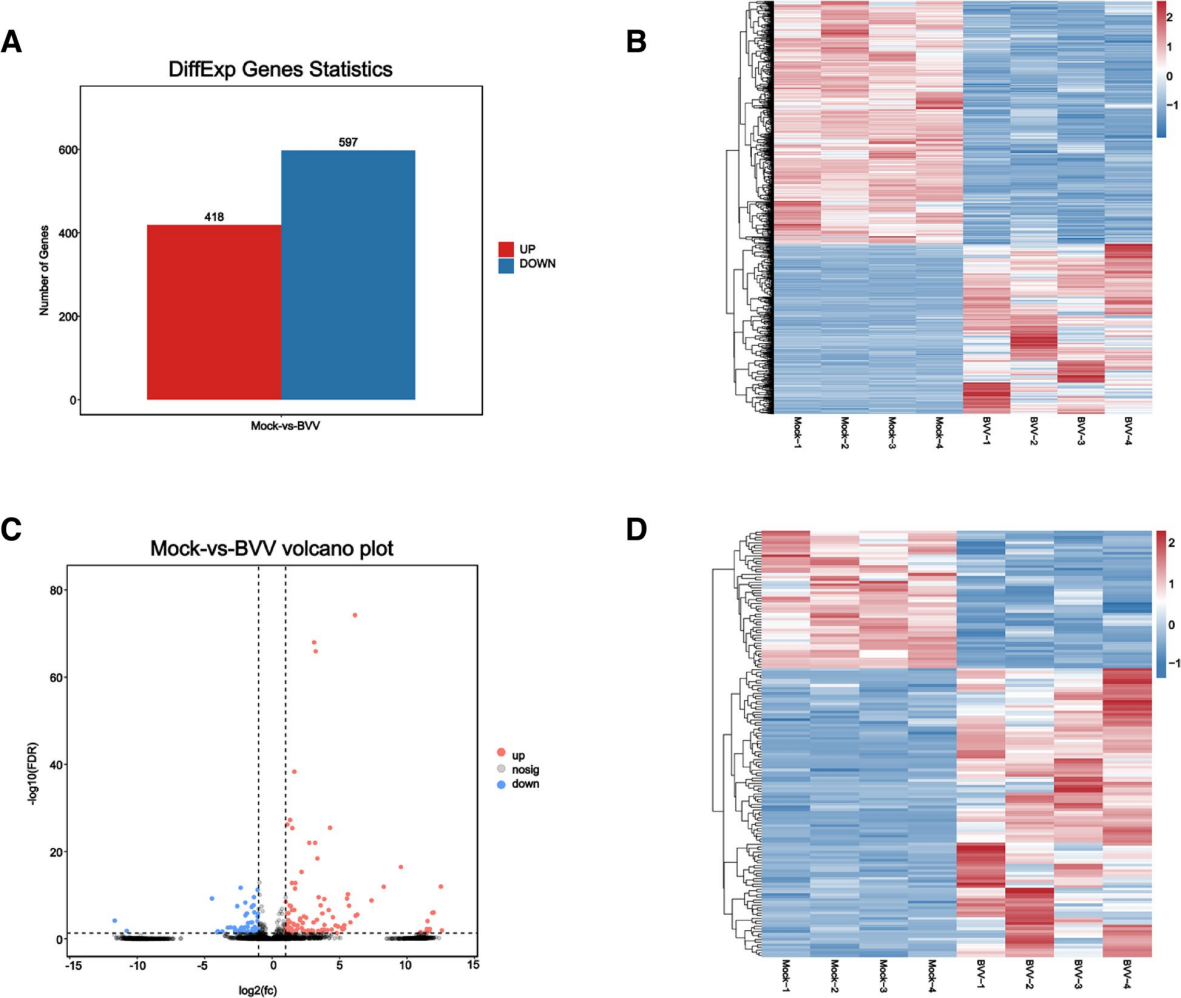
Basic differential expression analysis of the mRNAs and LncRNAs. Differentially expressed mRNA statistics (**A**). Heatmap of differentially expressed mRNAs (**B**). Volcano plot of global DEGs in comparison groups, Mock VS BVV. (**C**). Heatmap of global DEGs in comparison groups, Mock VS BVV. (**D**)

By analyzing the experimental and control groups with q-value ≤ 0.05 and |Fold change|> 2 as the screening condition for significant difference, we found that there were 1015 mRNAs, of which 418 were upregulated and 597 were downregulated (Fig. 2A). The differentially expressed mRNAs were clustered and analyzed, and the heatmap showed that there were significant differences between the experimental and control groups (Fig. 2B, Supplemental Table S1). There were 161 differentially expressed lncRNAs, of which 109 were upregulated and 52 were downregulated (Fig. 2C). The differentially expressed lncRNAs were analyzed using hierarchical clustering analysis, and the heatmap showed that there were obvious self-segregating clusters in the test and control groups (Fig. 2D, Supplemental Table S1).

### GO annotation and KEGG enrichment analysis of the differentially expressed mRNAs

**Fig. 3:**
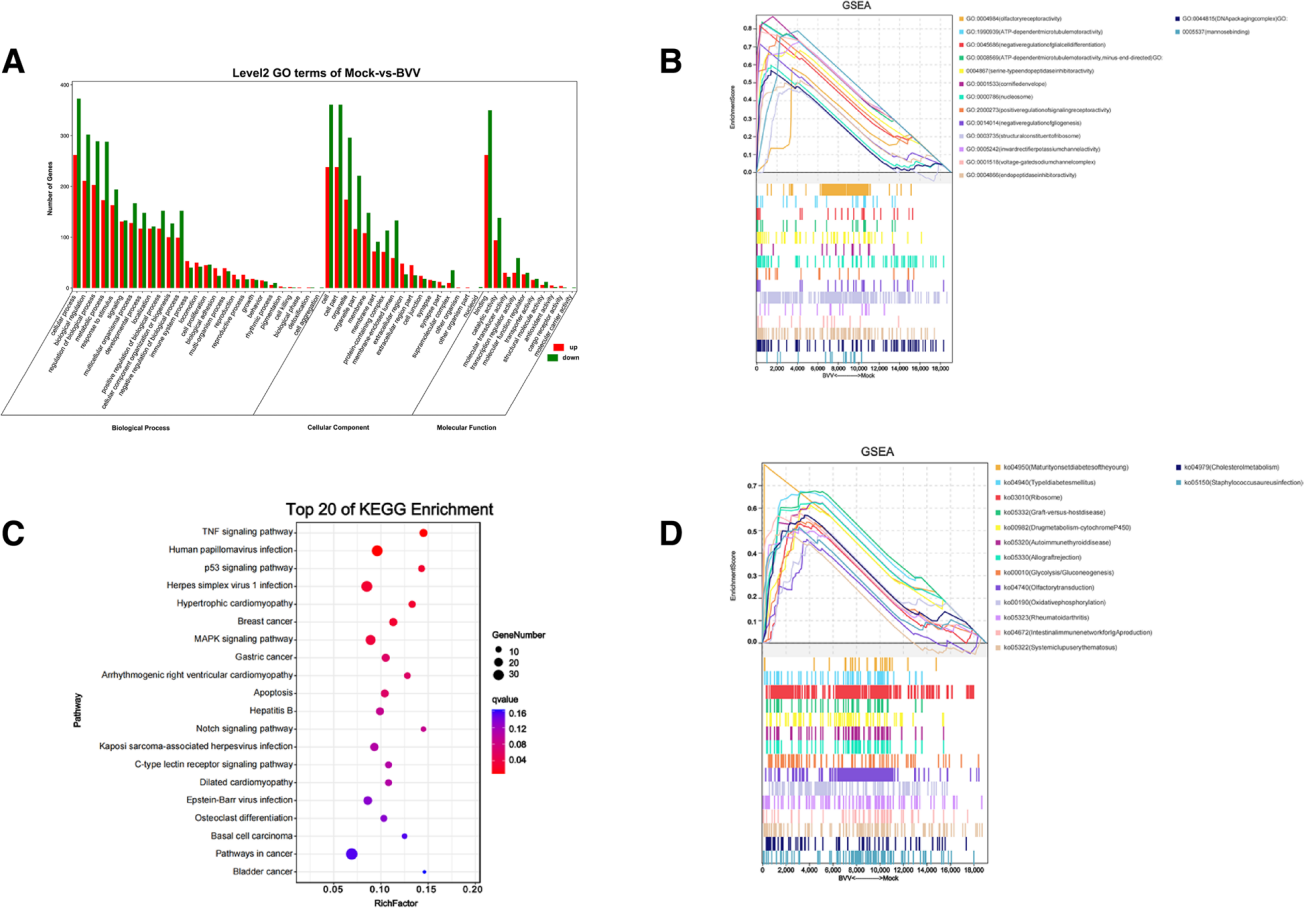
GO annotation and KEGG pathway enrichment analysis of the differentially expressed mRNAs. GO annotation classification of the differentially expressed genes (**A**). GSEA using the GO database (**B**). Significance bubble plots of the top 20 KEGG enrichment analyses (**C**). GSEAs using the KEGG database (**D**)

To provide a general description of the functions and pathways of the genes obtained through RNA-Seq analysis, we aligned these sequences with the GO and KEGG databases for functional annotation and classification. The GO enrichment analysis showed that the significantly different mRNAs in the mock vs. test group were mainly related to biological regulation, metabolic process, signaling, transcription regulator activity, and membrane (Fig. 3A, Supplemental Table S2). We performed gene set enrichment analysis using the GSEA software and MSigDB to identify whether a set of genes in specific GO terms showed significant differences in the two groups. The results showed that olfactory receptor activity, positive regulation of signaling receptor activity, inward rectifier potassium channel activity and voltage-gated sodium channel complex, and cell signaling pathway displayed significant differences in the two groups. Similarly, the pathways affecting protein synthesis and metabolism, including serine-type endopeptidase inhibitor activity, endopeptidase inhibitor activity, and structural constituent of ribosome, showed large differences. In addition, differences in the DNA packaging complex as well as the nucleosome pathway representing chromosome structure and function showed differences in the experimental and control groups (Fig. 3B, Supplemental Table S3).

The KEGG database contains a wealth of pathway information that contributes to the understanding of the biological functions of genes at the system level. The results of the KEGG enrichment analysis with q-value < 0.05 showed that the mRNAs with significant differences in the test group were mainly enriched in the TNF, MAPK, P53, and notch signaling pathways (Fig. 3C, Supplemental Table S4). The KEGG enrichment results of the GSEA showed that the signaling pathways related to energy metabolism and disease, such as Type I diabetes mellitus, graft-versus-host disease, autoimmune thyroid disease, glycolysis/gluconeogenesis, and oxidative phosphorylation, differed significantly in the two groups (Fig. 3D, Supplemental Table S3).

### GO annotation and KEGG enrichment analysis of the target genes of the lncRNAs

**Fig. 4:**
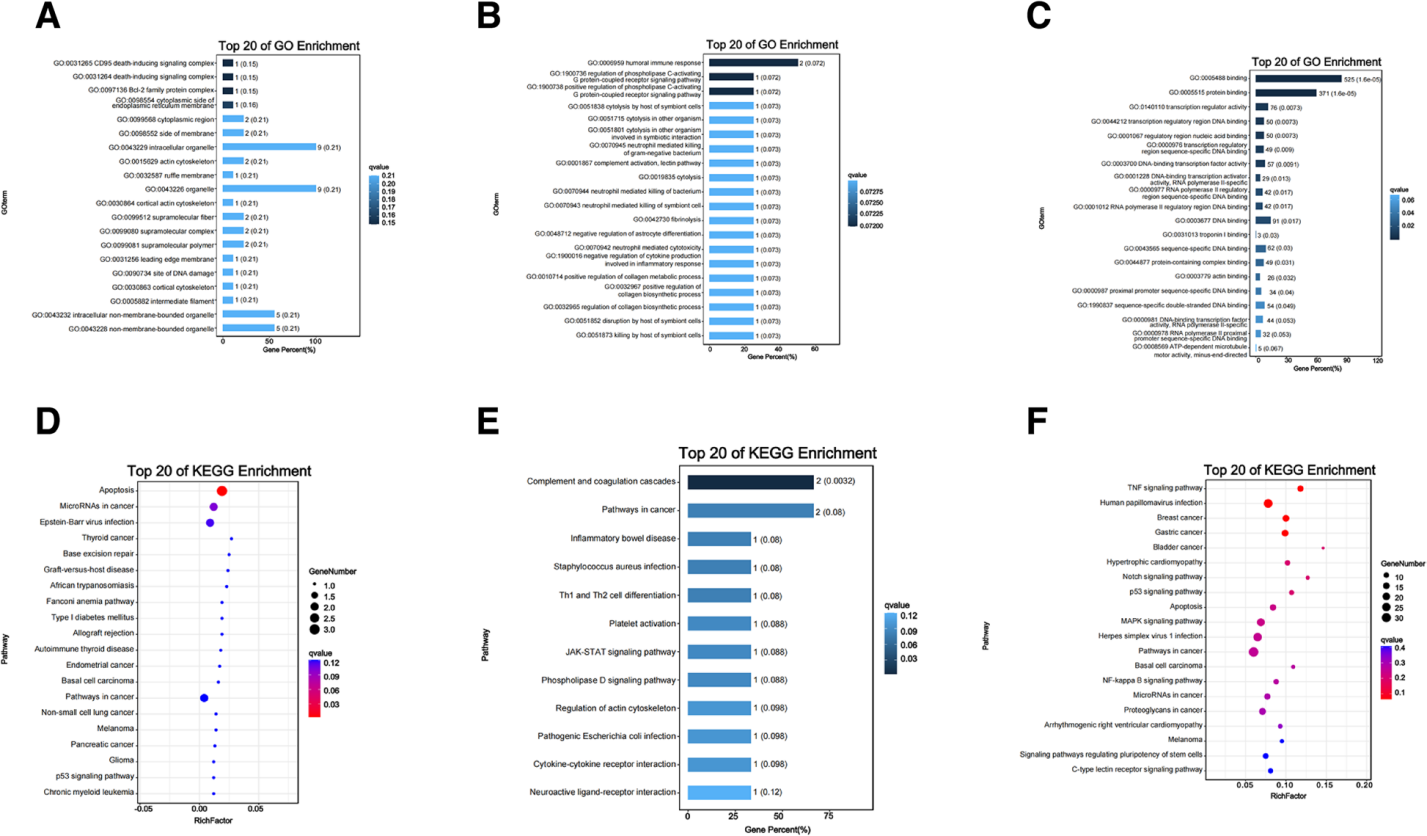
GO annotation and KEGG pathway analysis of the differentially expressed lncRNA targets. TP20 GO-enriched gradient of the cis- (**A**), antisense- (**B**), and trans-target genes (**C**). TP20 KEGG-enriched gradient of the cis- (**D**), antisense- (**E**), and trans-target genes (**F**)

The lncRNA target genes were analyzed based on GO annotations and KEGG enrichment. To gain a better understanding of the biological functions of the differential lncRNAs in the test and control groups, GO and KEGG enrichment analyses were performed on the lncRNA co-expressed target genes. GO analysis of the cis-target genes showed that the mRNAs co-expressed by the lncRNAs in VSV-infected BHK-21 cells were predominantly associated with the death-inducing signaling complex in cellular component (Fig. 4A, Supplemental Table S5). Antisense-targets were enriched for the humoral immune response, G protein-coupled receptor signaling pathway, and complement activation (lectin pathway) in biological processes (Fig. 4B, Supplemental Table S5). The trans-targets were enriched for protein binding, binding, transcription regulator activity, and DNA binding in molecular functions (Fig. 4C, Supplemental Table S5).

With q-value < 0.05 as the condition for the KEGG enrichment analysis, the results showed that the cis-target genes were mainly enriched in apoptosis, base excision repair, pathways in cancer, and the p53 signaling pathway (Fig. 4D, Supplemental Table S6). Antisense-targets genes were mainly enriched in the complement and coagulation cascades, pathways in cancer, JAK-STAT signaling pathway, and cytokine–cytokine receptor interaction (Fig. 4E, Supplemental Table S6). The trans-targets were mainly enriched in the TNF signaling pathway, pathways in cancer, p53 signaling pathway, MAPK signaling pathway, NF-kappa B signaling pathway, C-type lectin receptor signaling pathway, and apoptosis (Fig. 4F, Supplemental Table S6).

### lncRNAs and mRNAs co-expression network analysis

**Fig. 5:**
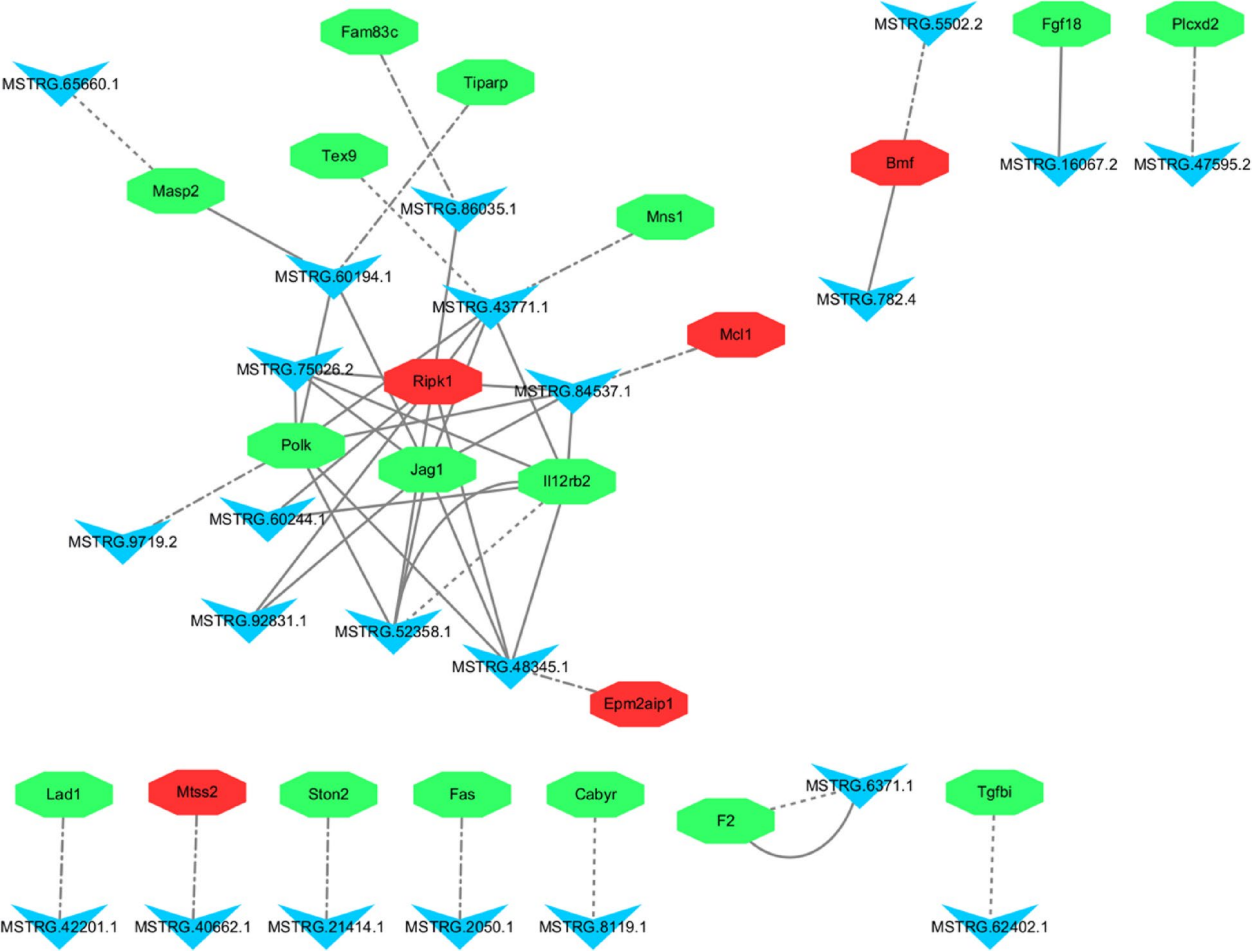
Network of the differentially expressed lncRNAs and lncRNA targets. The dashed-dotted, equal dashed, and solid lines represent cis-, antisense-, and trans-target genes, respectively. Red and green represent down- and upregulation, respectively. The circles and inverted triangles represent the target genes and lncRNAs, respectively

An lncRNA/mRNA co-expression network was constructed using 22 differentially expressed lncRNAs and 21 target genes. As shown in Fig. 5, some lncRNA targets were located in the center of the network, such as *Ripk1*, *Jag1*, *Polk*, *Tiparp*, and *Il12rb2*. Regarding viral infections, *Ripk1*-mediated innate immunity may play an important role in viral infections. The study by Xu et al. showed that SARS-CoV-2 could hijack the *Ripk1*-mediated host defense response to promote its own reproduction [12]. Likewise, in Getah virus (GETV)-infected Vero cells, *Tiparp* expression was significantly down-regulated, resulting in a significant increase in viral titer, suggesting that *Tiparp* overexpression significantly inhibited GETV replication. The host *Tiparp* is shown for the first time to be a limiting factor for GETV replication [13]. Several viruses, such as Herpes Simplex Virus type 2 and Human Immunodeficiency Virus, regulate the expression of *Fas* ligands upon infection of cells, resulting in programmed cell death, which may be a flexible mechanism for viruses to enhance replication and the immune evasion [14, 15].

Network modeling revealed that immune-related lncRNA targets were co-expressed with the lncRNAs, suggesting that the lncRNAs and mRNAs are mutually regulated in immune processes, such as viral infection.

### RT-qPCR validation of the differentially expressed genes

**Fig. 6:**
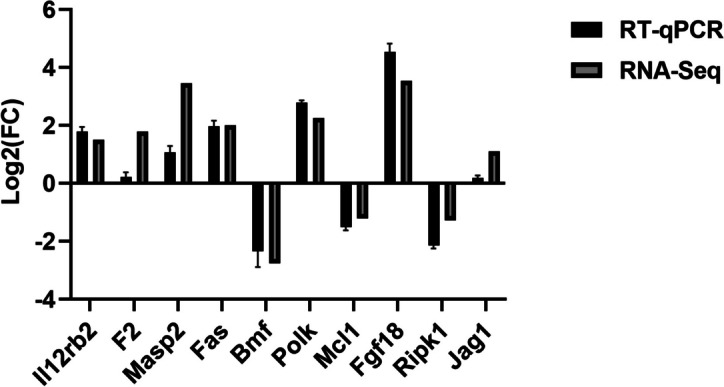
RT-qPCR verification of the differentially expressed genes. Statistical comparisons were made using GraphPad Prism 10.0. Values (RT-qPCR) shown were mean with SD

In this experiment, RNA-Seq analysis was used to analyze a total of 161 differential lncRNAs and 1015 differential mRNAs. Ten differentially expressed genes, namely *Il12rb2*, *F2*, *Masp2*, *Fas*, *Bmf*, *Polk*, *Fgf18*, *Jag1*, *Ripk1*, and *Mcl1*, were randomly selected and detected through quantitative real-time PCR. The expression of *Il12rb2*, *F2*, *Masp2*, *Fas*, *Polk*, *Fgf18*, and *Jag1* was upregulated. The expression of *Bmf*, *Mcl1*, and *Ripk1* was downregulated (Fig. 6). The validation results of the RT-qPCR were consistent with the RNA-Seq results, proving the reliability of the results of this experiment.

## Discussion

Vesicular stomatitis is a disease of hoofed animals characterized by blistering lesions on the oral mucosa and feet. VSV infection can cause harm and economic loss to livestock farming. However, VSV can be extremely useful in medical applications. As mentioned previously, many properties make VSVs excellent vaccine carriers. In addition, it has shown encouraging antitumor activity in a variety of human cancer types. VSV is particularly attractive as a tumorolytic agent due to its broad tropism, rapid replication kinetics, and susceptibility to genetic manipulation. In addition, VSV-induced tumor lysis can trigger potent antitumor cytotoxic T-cell responses to viral proteins and tumor-associated antigens, resulting in durable antitumor effects. Because of this multifaceted immunomodulatory property, VSV has been extensively investigated for immunoviral therapy alone or in combination with other anticancer modalities such as immune checkpoint blockade [16]. The transcriptome analysis of VSV-infected BHK-21 cells in this study provides insights into the mechanisms of VSV–host interactions as well as a theoretical basis for subsequent disease detection, vaccine and drug development, and more in-depth research.

In this study, RNA-Seq was performed on BHK-21 cells infected with VSV for 24 h. To understand the regulatory functions of these lncRNAs, the differential lncRNAs co-expression mRNAs were predicted and functionally analyzed, and it was found that the lncRNAs co-expression mRNAs were mainly enriched in the TNF, MAPK, and P53 signaling pathways. They were also related to the KEGG pathways such as cancer: overview, infectious disease: viral, immune system, and cell growth and death.

In this study, 10 differentially expressed genes were randomly selected for validation, and the results showed that the expression of all 10 genes was upregulated or downregulated, suggesting that they may play an important role in the process of viral infection. *FGF18* selectively binds to *FGFR3*; it is an essential mitogen for embryonic limb development; and it is required for lung development and disease. Studies have shown that *FGF18* has multiple organ developmental and injury repair effects [17]. *FGF18* expression was significantly increased in VSV-infected hosts, suggesting that viral infection influences cell fate. *Ripk1* plays an important role in pathways such as the TNF, MAPK, and NF-kappa B signaling pathways, and it may be involved in apoptotic processes. Targeting *Ripk1* in the treatment of neurological diseases may help inhibit multiple cell death pathways and ameliorate neuroinflammation [18]. In our study, significant changes in *Ripk1* expression may be involved in the immune defense of the cells. In addition, *Il12rb2*, *F2*, and *Masp2* are involved in the activities of the cellular immune system and play important roles in viral infections and cancer [19–21]. Both *Il12rb2* and *F2* may be implicated in innate immunity. Mannan-binding lectin–associated serine protease 2 co-activates the lectin pathway of the complement in response to several viral infections [21]. *Fas* (*CD95/Apo-1*) is a member of the "death receptor" family, which is a group of cell surface proteins that trigger apoptosis by binding to their natural ligands [22]. The p53 signaling pathway, in which *Fas* is engaged, is associated with cell cycle arrest, cellular senescence, and apoptosis. The TNF signaling pathway is closely related to inflammation as well as cancer, and it also activates various pathways, including NFκB and MAPK. It has been shown that *BMF* mediates fetal oocyte loss in mice [23]. In diabetic mice, inhibition of *BMF* transcription by hnRNP F is an important mechanism by which insulin protects diabetic RPTC from apoptosis [24], suggesting that *BMF* may play an important role in the apoptotic process. *POLK*, which encodes the specialized transposable synthesis DNA polymerase κ, is known to perform precise DNA synthesis on microsatellites. Transcriptional regulation of *POLK* involves some of the p53 tumor suppressors [25]. It has been shown that *Polk* (-/-) mice have a significantly increased frequency of mutations in the kidneys, liver, and lungs [26]. These results suggest that *Pol* kappa is required for accurate translational DNA synthesis and that significant changes in its expression may be associated with the activation of the cancer pathway. *MCL1* is a pro-survival (antiapoptotic) protein commonly expressed in hematological tumors and plays an important role in their biology, either through dysregulation or due to its intrinsic importance to the cells of origin of malignant tumors [27]. *Jagged1* (*JAG1*) is one of five cell-surface ligands that function primarily in the highly conserved notch signaling pathway. Variations in *JAG1* are associated with several types of cancers, including breast and adrenocortical cancers [28].

Seropositivity to VSV antibodies is generally low in the population; thus, pre-immunization against vectors is rare, and viral sequences are unlikely to integrate into the host genome [1]. Therefore, the future of VSV as a vaccine carrier is very promising. In preclinical studies, it has been shown that VSV-based vaccines induce strong humoral and cellular immune responses after vaccination [29]. Several vaccines have previously been developed based on recombinant VSV with good protection. For example, recombinant VSV expressing measles virus (MV) hemagglutinin (VSV-h) induces high titers of MV-neutralizing antibodies in the presence of MV-specific antibodies and provides good protection against subsequent MV attacks [30]. From the results of this experiment, VSV infection activated the cellular MAPK signaling pathway, P53 signaling pathway, TNF signaling pathway, and other pathways related to the activation of the immune system as well as pathways related to cancer and apoptosis. This provides evidence that VSV is an excellent vaccine carrier.

To sum up, in our study, we performed functional analyses of differential lncRNAs and mRNAs and differential lncRNA and mRNA combination analyses; screened potential candidate lncRNAs and target genes in VSV infections; and analyzed the functions of these target genes as well as the pathways in which they are located. The results suggest that VSV infection activates TNF, MAPK, NF-kappaB and other immune-related pathways. Among them, some genes in these pathways were also up- or down-regulated, including *Ripk1*, *Il12rb2*, *Masp2*, etc., in which reveal that VSV infection causes alterations in the host metabolic network.

Our study revealed the process of physiological changes in host cells during VSV infection, which contributes to further understanding of the pathogenesis of the virus and provides a basis for the next steps in detection, prevention, and treatment, as well as a direction for further research on the interaction between VSV and the host. Pan et al. showed that the eukaryotic translation initiation factor 3, subunit i (eIF3i) may affect VSV growth by modulating the host antiviral response in HeLa cells [31]. Kueck et al. also found that a specific antiviral protein, TRIM69, interacts with and inhibits the function of a particular phosphoprotein (P) component of the VSV transcriptional machinery, thereby preventing the synthesis of viral messenger RNAs [32]. All of these results illustrate the mechanism of VSV-host interactions at the molecular level. Furthermore, our results reveal the interaction between VSV and host can be explored on this basis at the gene level. More importantly, VSV has a wide range of applications as a molecular tool and vaccine carrier. The transcriptome sequencing results showed that VSV can effectively activate the immune system of host cells, which may mean that a live viral vector vaccine using VSV as a carrier can effectively stimulate the body to produce antibodies. Our laboratory has established a mature reverse genetic system for VSV, and the results of this experiment provide a theoretical basis for the construction of future vaccines using VSV as a vector. However, our results are still based on a cellular level, which need further animal experiments to verify at the individual level.

## Conclusions

In this study, we performed RNA-Seq on VSV-infected BHK-21 cells. The differential genes enriched were mainly connected to pathways related to apoptosis and tumor. Our results indicate that VSV infection causes alterations in the host metabolic network, which provides unique insights for further studies on the mechanisms of VSV–host interactions as well as a basis for the development of potent drugs and vaccines for VSV. More importantly, VSV activated pathways related to the cellular immune system, cancer, and apoptosis, providing evidence that VSV can be used as a live virus vaccine vector. Our results also provide a theoretical basis for studying VSV infection at the gene level, pointing the way to deeper theoretical studies.

## Methods

### Virus and cells

BHK-21 was purchased from ATCC (https://www.atcc.org/). The Indiana strain of VSV was from our laboratory.

### Growth curves of VSV

The growth curve of VSV at an MOI of 1 was determined. Briefly, BHK-21 cells (2 × 10^6^ cells/well) were inoculated in six-well cell culture plates and cultured in DMEM containing 10% fetal bovine serum. After 12 h, BHK-21 cells were inoculated with VSV at 1.0 MOI and incubated at 37 °C with 5% CO_2_. The supernatants were collected in TRIzol solution at 4, 8, 12, 24, 36, and 48 h. Three biological replicates were set up for each group. The growth curve of VSV was determined using the following method: 1) Quantitative analysis of the viral genome was performed using reverse transcription real-time quantitative PCR; 2) the titer of the virus in the above supernatant was determined using the plaque assays, and growth curves were plotted.

### Sample collection

BHK-21 cells were inoculated in six-well cell culture plates using DMEM containing 10% fetal bovine serum and cultured at 37 °C with 5% CO_2_ for 12 h until the monolayer of cells grew to 95% confluence. BHK-21 cells were inoculated with 1.0 MOI of VSV, incubated for 1 h, supplemented 2 ml of DMEM containing 1% fetal bovine serum, and then maintaining at 37C with 5% CO2. Samples were collected 24 h after infection according to the growth curve of VSV. The experiment was performed in four biological replicates.

### RNA extraction, library construction, and sequencing

Total RNA was extracted using the TRIzol reagent (Invitrogen, Carlsbad, CA, USA) according to the manufacturer's protocol. RNA quality was assessed on an Agilent 2100 Bioanalyzer (Agilent Technologies, Palo Alto, CA, USA) and checked using RNase-free agarose gel electrophoresis. After total RNA was extracted, rRNAs were removed to retain mRNAs and lncRNAs. Then, the enriched mRNA and lncRNA were fragmented into short fragments using fragmentation buffer and reversely transcribed into cDNA using the NEBNext Ultra RNA Library Prep Kit for Illumina (NEB #7530, New England Biolabs, Ipswich, MA, USA). The purified double-stranded cDNA fragments were end-repaired, and A-nucleotide overhangs were added, followed by ligation to Illumina sequencing adapters. The ligation reaction was purified with AMPure XP Beads (1.0 x) and PCR-amplified. The resulting cDNA library was sequenced using the Illumina Novaseq6000 platform by Gene Denovo Biotechnology Co.

### Reference genome mapping and transcriptome assembly

To obtain clean reads, fastp (version 0.18.0) was used to filter the raw reads. The process included removing reads containing adapters, removing reads containing more than 10% of unknown nucleotides, and removing low-quality reads containing more than 50% of low-quality (Q-value ≤ 20) bases. Bowtie2 (version 2.2.8) was used for mapping the clean reads to the ribosomal RNA database. The rRNA mapped reads then will be removed. The remaining clean reads of each sample were assembled using StringTie (version 1.3.1) in a reference-based approach.

An index of the reference genome was built, and paired-end clean reads were mapped to the reference genome, *Mesocricetus auratus*, using HISAT (version 2.2.4), and other parameters set to default.

### Identification of potential lncRNA candidates

Three softwares CNCI (version 2.0), CPC (version 0.9-r2) and FEELNC (version 0.2) were used to assess the protein-coding potential of novel transcripts by default parameters. The intersection of both non-protein-coding potential results were chosen as lncRNAs while they met the conditions of length > 200 bp and exon number > 2.

### Relationship analysis of the samples

Correlation analysis was performed using the R statistical software. Principal component analysis (PCA) was performed with the R package gmodels (http://www.rproject.org/). PCA is a statistical procedure that converts hundreds of thousands of correlated variables (transcripts expression) into a set of values of linearly uncorrelated variables called principal components. PCA is largely used to reveal the relationship of the samples.

### Analysis of expression

The RSEM software was used to calculate the expression of the transcription region. The fragment per kilobase of transcript per million mapped reads value was calculated to quantify its expression abundance and variations. The DESeq2 software was used to conduct differential expression analysis for the two different groups, in which the statistical significance was set at a false discovery rate (FDR)-adjusted *p*-value (padj ≤ 0.05) and |Log2Foldchange|> 2.

### Gene function enrichment analysis

All differentially expressed genes (DEGs) were mapped to Gene Ontology (GO) terms in the Gene Ontology database (http://www.geneontology.org/). Significantly enriched GO terms in DEGs comparing to the genome background were defined by hypergeometric test. The calculated p-values were subjected to FDR Correction, with FDR ≤ 0.05 as the threshold. The p-value was calculated using the R phyper hypergeometric test and the qvalue (version 2.2.2) was used to calculate the FDR.

KEGG (https://www.kegg.jp) is a manually managed database resource that integrates various biological objects [33]. KEGG links genomic with higher-order functional information, i.e., the information in the PATHWAY database [34]. The purpose of KEGG pathway maps is to establish links from genes in the genome to gene products in the pathway [35]. Each pathway of KEGG was analyzed for enrichment using the hypergeometric test. The calculation formula was the same as that in the GO analysis. Pathways meeting this condition were defined as significantly enriched pathways in the DEGs.

### Gene set enrichment analysis (GSEA)

We performed gene set enrichment analysis using the GSEA software and MSigDB to identify whether a set of genes in specific. Briefly, we input the gene expression matrix and ranked genes using the SignaltoNoise normalization method. Enrichment scores and p-values were calculated with the default parameters.

### Gene function enrichment analysis of differentially expressed lncRNA targets

LncRNAs regulate target genes by cis-, antisense- and trans-regulation. The three modes of regulation follow their own methods of target gene prediction. The software RNAplex (version 0.2) was used to predict the antisense-targets. LncRNAs in less than 100 kb up or down stream of a gene were assumed to be cis-regulators. As for lncRNA trans-regulation analysis, Pearson correlation > 0.999 was used as a condition to screen target genes. The cis-, antisense- and tran-targets that matched the screening criteria were subjected to GO, as well as KEGG enrichment analysis by referring to Gene function enrichment analysis.

### Construction of lncRNA/mRNA networks

The relevant descriptions of the target genes were available by searching the NCBI database through GeneBank accession number. To infer the functions of the differentially expressed lncRNAs and their target genes, we constructed a network based on lncRNAs and mRNAs in Cytoscape (version 3.1.1).

### Validation of transcriptome sequencing results

Reverse transcription real-time quantitative PCR (RT-qPCR) was performed to validate the genes identified by transcriptome sequencing. Ten random DEGs were selected for RT-qPCR validation. Total RNA extraction was performed using the TRIzol reagent according to the manufacturer's instructions. M-MLV reverse transcriptase (Bao Bioengineering Co., Ltd., Dalian, China) was used for cDNA synthesis. Sequence-specific primers were designed for the randomly selected genes using the SnapGene software (Table 2). RT-qPCR was performed using the Roche LightCycler® 480II Real-Time Fluorescent Quantitative PCR System (Roche, Switzerland). RT-qPCR was performed in a 10 μL reaction volume containing 5 μL of TB Green® Premix Ex Taq™ II (Tli RnaseH Plus) (Bao Bioengineering Co., Ltd., Dalian, China), 0.3 μL of upstream and downstream primers (10 μM), 1 μL of cDNA template, and 3.4 μL of ddH_2_O. The following reaction profile was used: 95 °C for 5 min, followed by 40 cycles of 95 °C for 10 s, and 60 °C for 30 s. Melting curve analysis was performed to validate specific amplification. The *β-actin* gene was used as an endogenous reference gene. RT-qPCR was performed in a 384-well plate, and each biological replicate was tested in triplicate. The relative expression values of the selected genes were calculated using the 2^−ΔΔCt^ method and normalized against the expression levels of the *β-actin* gene.

**Table 2:** Primers used for RT-qPCR in this study

Name	Primer sequence	Name	Primer sequence
Il12rb2-F	CATTGCCTCCAGTCCACAACCTAT	Polk-R	AGAATGGTGATGGTGTGCGTCTT
Il12rb2-R	ATGTGTTGATGCTCCTCCAGTTCT	Mcl1-F	AGTGGCTACAGGATTGTGGCTAAC
F2-F	GGAACGGGAAATATGGCTTCTA	Mcl1-R	CTACAGGAGTGGAGTGACGGACT
F2-R	GGAACGGGAAATATGGCTTCTA	Fgf18-F	TCTACTGCTGTGCTTCCAGGTTCA
Masp2-F	CTTATTACTGGGTGAAACTATGGCA	Fgf18-R	GCAACTGCTTCCGACTCACATCAT
Masp2-R	CAGGCTTGGGTGTAGTCAGGT	Ripk1-F	CGAGTTGTGGCTGAACCTGTATGA
Fas-F	CTGCTGAGAGGTGGAGACTTGG	Ripk1-R	ACCTACCAATGACTGGCTCCGTAT
Fas-R	ATGGTTGCCTTGGAGATGCTATCA	Jag1-F	AGCCTGTGAGCCTTCCTTGTCT
Bmf-F	GCAATGCTGGCTACAGGCTTCC	Jag1-R	GACGCCTCTGAACTCTGACTTCTG
Bmf-R	CTCGGTTCTGCTGGTGTTGTTGT	β-actin-F	CTGTGCTATGTTGCCCTGGACTTC
Polk-F	CAGGAAGGTGTCAGTCTGGAAGC	β-actin-R	CCGCTCGTTGCCAATGGTGAT

### Statistical analysis

All data were analyzed using IBM SPSS Statistics 26.0. All data are presented as the mean ± SD. The t tests were performed to compare means, and *P* < 0.05 was considered statistically significant.

### Supplementary Information

**Additional file 1. **

**Additional file 2. **

**Additional file 3. **

**Additional file 4. **

**Additional file 5. **

**Additional file 6. **

## Data Availability

Data is available at the Sequence Read Archive (SRP465591) with the site https://dataview.ncbi.nlm.nih.gov/object/PRJNA1025338?reviewer=16snf0n1targvkujcsh52ubsca. The datasets used and analysed during the current study available from the corresponding author on reasonable request.

## Abbreviations

LncRNAs: Long noncoding RNAs
TNF: Tumor necrosis factor
MAPK: The mitogen-activated protein kinase cascade
NF-kappa B: Nuclear factor kappa-B
Il12rb2: Interleukin-12 receptor subunit beta-2
F2: Coagulation factor II
Masp2: Mannan binding lectin serine peptidase 2
Mcl1: Myeloid cell leukemia sequence 1
Fgf18: Fibroblast growth factor 18
Ripk1: Receptor interacting serine/threonine kinase 1
Fas: Fas cell surface death receptor
BMF: Bcl2 modifying factor
POLK: DNA polymerase kappa
JAG1: Jagged canonical Notch ligand 1
